# Surfactant-Modulation of the Cationic-Polymer-Induced Aggregation of Anionic Particulate Dispersions

**DOI:** 10.3390/polym12020287

**Published:** 2020-02-01

**Authors:** Wasiu Abdullahi, Martin Crossman, Peter Charles Griffiths

**Affiliations:** 1 School of Science, Faculty of Engineering and Science, University of Greenwich, Chatham Maritime, Kent ME4 4TB, UK; W.O.Abdullahi@greenwich.ac.uk; 2 Unilever Research, Port Sunlight, Quarry Road East, Bebington, Wirral CH63 3JW, UK; Martin.Crossman@unilever.com

**Keywords:** flocculation, polyelectrolyte, bridging flocculation, solvent relaxation, charge-charge interactions, polyelectrolyte-surfactant interactions

## Abstract

Commodity formulations contain many chemically distinct components and their mutual interactions define the beneficial characteristics of the formulation. Mixing oppositely charged polymers and surfactants invariably induces macroscopic phase separation, to a degree dependent on the prevailing polymer and surface charge densities, and the interaction can be modulated by added ionic surfactants. Here, it is shown that a general universality exists between the charge present on a series of cationic-modified cellulose polymers—the charge being controlled either by the degree of cationic modification of the polymer itself or through the subsequent level of anionic surfactant binding—and its capacity to remove anionic colloidal material from solution, be that silica particles or polystyrene-butadiene lattices. Particulate material not removed from solution bears no adsorbed polymer, i.e., the particle surface is bare. Addition of nonionic surfactant does not negate this universality, implying that the nonionic surfactant is largely a spectator molecule or structure (micelle) in these systems, and that the dominant force is an electrostatic one.

## 1. Introduction

The adsorption of polymers to particulate surfaces continues to attract much attention due to its diverse industrial relevance, and depending on the specific application, the role of the polymer may be to promote stabilization of the dispersion, to induce flocculation or otherwise destabilize the dispersion, or a combination of both, dependent on the polymer concentration [1,2,3,4,5]. The adsorption of neutral homopolymers and copolymers onto surfaces is driven by a complex and subtle interplay between molecular weight and concentration-dependent enthalpic and entropic factors in defining the amount of adsorbed polymer, and inter alia its conformation at the particle surface [6,7]. The presence of charge on the polymer leads to additional contributions to the thermodynamics of adsorption when exposed to both oppositely and like-charged surfaces, and usually, the electrostatic force dominates [8,9,10]. The structure of the resultant polyelectrolyte-particle complex is defined by the polyelectrolyte and surface charge densities, polymer molecular weight and architecture, salt concentration/ionic strength of the medium, nonelectrostatic polyelectrolyte-surface interactions and the quality of the solvent [11,12,13].

Flocculation-inducing adsorption of polyelectrolytes to particle surfaces has been studied in some detail (e.g., [14,15,16,17,18]), and generally, there are three dominant mechanisms dependent on the molecular weight and/or charge density of the polyelectrolyte: (i) neutralization of the charge on the particle surface leading to attractive van der Waals-driven aggregation; (ii) interparticle bridging by low/medium charge density, high-molecular-weight polymers; and (iii) attraction between uncoated surfaces and oppositely charged patches on adjacent particles formed by the localised adsorption of high charge density, low-molecular-weight polyelectrolytes [19,20,21].

The impact of added surfactant on polyelectrolyte-induced flocculation of particle dispersions has also been explored. For example, Barany and Skvarla [22] studied the adsorption of both cationic and anionic high-molecular-weight, high-charge-density polyelectrolytes to carboxylated polystyrene particles in the presence of hexadecyl pyridinium bromide (HPB) or anionic surfactant sodium dodecyl sulfate (SDS). Taubaeva et al. [23] extended that study to bentonite dispersions and showed that addition of mixtures of cationic surfactants and polyelectrolyte induces a substantial reduction in the negative charge on the particles, including a reversal of the sign of their charge, whereas, the addition of anionic surfactant/polyelectrolyte mixtures leads to a substantial increase in the particle charge. Besra et al. [24] studied the flocculation of kaolin suspensions with high-molecular-weight polyacrylamide flocculants (cationic, anionic and nonionic) of varying ionic character in the presence of cetyl trimethyl ammonium bromide (CTAB), SDS, and Triton-X 100. Karlson et al. [25] explored the interaction of two cellulosic polymers, ethyl hydroxyethyl cellulose (EHEC) and hydrophobically modified ethyl hydroxyethyl cellulose (HM-EHEC), with both polystyrene latex and silica particles, in the presence of SDS, CTAB, and octaethylene glycol mono n-dodecyl ether (C_12_E_8_). Lele and Tilton [26] examined the depletion and structural forces operating between silica spheres and plates in solutions containing the anionic polyelectrolyte sodium polyacrylate (Na-PAA) and SDS, and observed situations where the depletion attraction was synergistically enhanced or antagonistically weakened and where one species was the dominant depletant and indistinguishable from its single component behavior. Matusiak and Grządka [27] studied the efficiency of cationic starch to flocculate silica dispersions in the presence of anionic surfactants, highlighting the switch from bridging flocculation at low-polymer concentrations to a depletion mechanism at higher concentrations, surfactant-induced enhancement in polymer adsorption at low-surfactant concentrations and polymer-surfactant complex adsorption at higher concentrations.

Cationic hydroxyethylcellulose (cat-HEC) polymers, produced by quaternization of hydroxyethylcellulose, are widely used in water-based formulations, since the cationic groups along the polymer backbone lead to significant increases in viscosity and offer control over colloidal stability [28]. Extending the work of Shubin [29], Terada et al. examined the effect of the anionic surfactant SDS on the adsorption of cationic hydroxyethyl celluloses to silica surfaces (in the presence of 10 mM NaCl) using null ellipsometry. They showed that the surfactant-concentration-dependent associative binding of the surfactant to the polymer defined the interfacial behaviour, that maximum adsorption was observed in the low-surfactant concentration one-phase region, whilst higher SDS concentrations led to desorption of the complex from the surface [30].

This study focuses on the interaction of cat-HEC with anionic particles, in the presence of the anionic surfactant sodium dodecyl sulfate (SDS) and its mixture with the nonionic surfactant hexaethylene glycol monododecyl ether (C_12_E_6_), to explore how the degree of apparent cationic charge on the polymer defines its ability to adsorb to silica and thus, to understand how the surfactant(s) modulate(s) the polymer-particle interaction.

## 2. Materials and Methods

Four cationic cellulosic polymers have been explored in this work, in concert with an anionic silica dispersion (Ludox TM-50, particle size 22 (+/–2) nm, surface area 140 m^2^·g^−1^) and a hydrophilic, anionic polystyrene-polybutadiene latex (particle size 100 (+/–5) nm, surface area 53 m^2^·g^−1^). The majority of samples have been prepared in deionized water, with the particle dispersions being extensively dialyzed prior to use. Sodium dodecyl sulfate (SDS) and hexaethylene glycol monododecyl ether (C_12_E_6_) were used as received (Sigma-Aldrich). For both surfactants, the surface tension data showed no minima characteristic of significant levels of impurity, and the mass spectrometry and NMR data were consistent with the accepted structures.

**Scheme 1:**
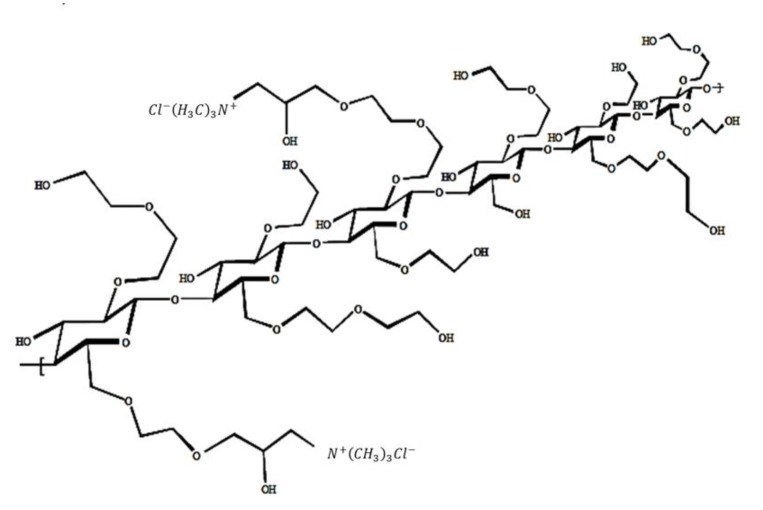
Generic structure of quaternary ammonium salts of hydroxyethyl cellulose polymers. Adapted from ref [31].

The cationic cellulosic polymers are commercial in nature, Scheme 1 (Dow Chemical Company), and will be referred to by their nominal degrees of modification, expressed in terms of the nitrogen content; N = 0.5% (±0.1)%, N = 0.95% (±0.15)% and N = 1.8% (±0.2)%. The parent polymer has also been included for comparison, and is labelled N = 0. The manufacturer’s literature suggests these polymers have molecular weights in the vicinity of 500,000 g·mol^−1^.

Order of mixing is known to be a significant factor in these systems, and therefore all samples were prepared by adding polymer stock solutions (or their dilutions) to the particulate dispersions, and where surfactant (SDS or C_12_E_6_) is additionally present, this was added to the polymer stock solution prior to adding to the silica. The importance of order of addition highlights the nonequilibrium effects often observed in these systems.

The solvent-relaxation measurements were determined on a Zigo Acorn Drop bench-top NMR spectrometer operating at 13.0 MHz. Samples were equilibrated at 25 °C. The instrument’s in-built configuration macros and data-analysis routines were used to optimize and obtain the experimental relaxation rates. All results are the average of at least triplicate measurements and frequent duplicate samples.

## 3. Results and Discussion

**Figure 1:**
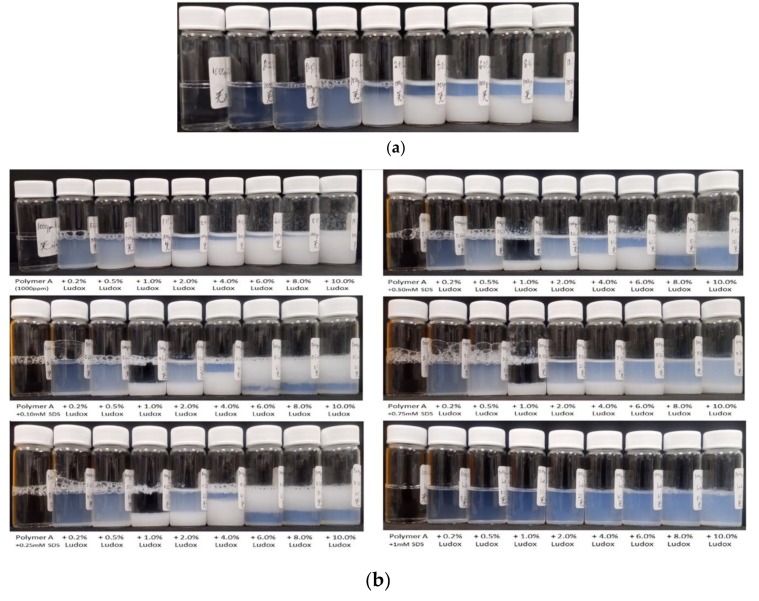

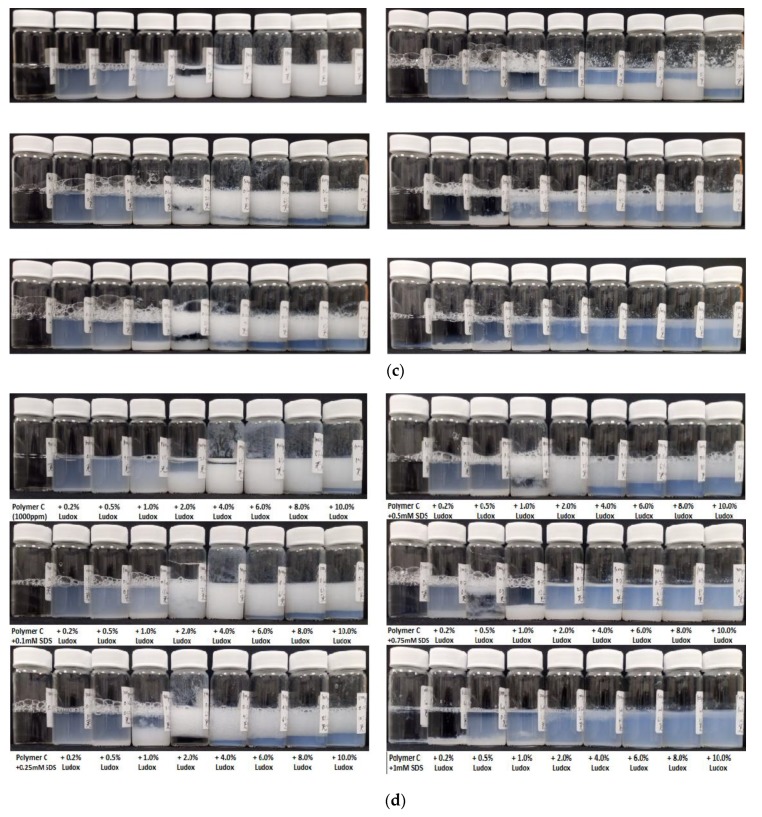
(**a**) Visual appearance of silica/polymer mixtures at 25 °C. The (uncharged) polymer (N = 0%) concentration is 1000 ppm throughout, the silica concentration is increased from 0 to 10 wt %, viz., 0 (left), 0.2, 0.5, 1.0, 2.0, 4.0, 6.0, 8.0, 10.0 (right). Photographs were taken approximately 2 days after preparation. (**b**) Visual appearance of silica/polymer/sodium dodecyl sulfate (SDS) mixtures at 25 °C. The polymer (N = 0.5%) concentration is 1000 ppm throughout, the silica concentration is increased from 0 to 10 wt % and SDS is 0, 0.1 and 0.25 mM (left-hand column), 0.50, 0.75 and 1.0 mM (right-hand column). Photographs were taken approximately 2 days after preparation. (**c**) Visual appearance of silica/polymer/SDS mixtures at 25 °C. The polymer (N = 0.95%) concentration is 1000 ppm throughout, the silica concentration is increased from 0 to 10 wt % and SDS is 0, 0.1 and 0.25 mM (left-hand column), 0.50, 0.75 and 1.0 mM (right-hand column). Photographs were taken approximately 2 days after preparation. (**d**) Visual appearance of silica/polymer/SDS mixtures at 25 °C. The polymer (N = 1.8%) concentration is 1000 ppm throughout, the silica concentration is increased from 0 to 10 wt % and SDS is 0, 0.1 and 0.25 mM (left-hand column), 0.50, 0.75 and 1.0 mM (right-hand column). Photographs were taken approximately 2 days after preparation.

**Figure 2:**
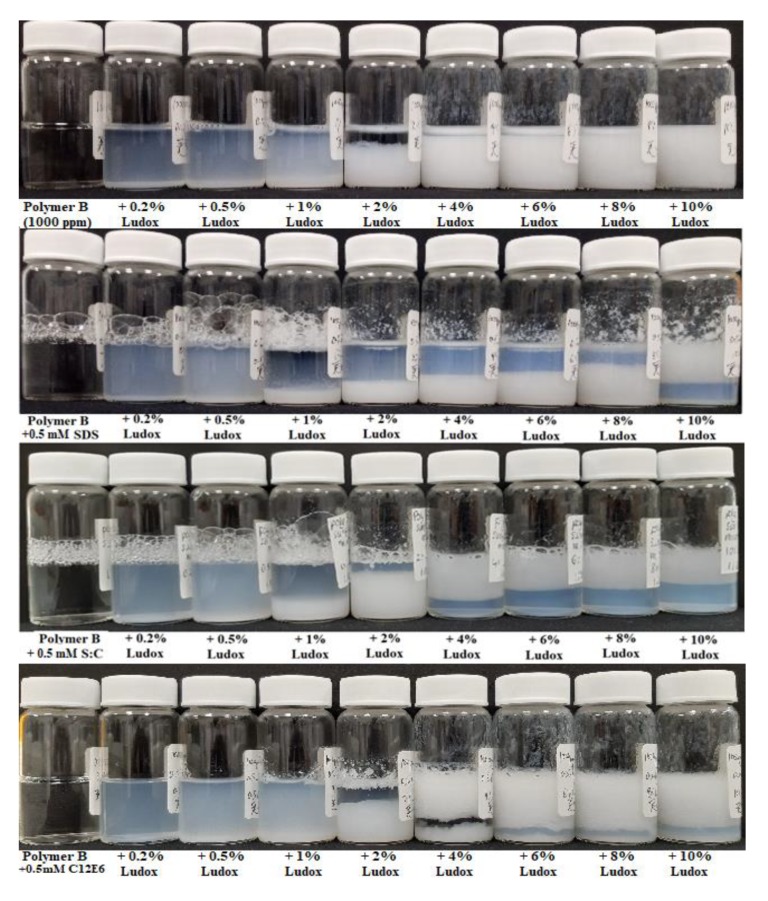
Visual appearance of silica/polymer/SDS/C_12_E_6_ mixtures at 25 °C. The polymer (N = 0.95%) concentration is 1000 ppm throughout, the silica concentration is increased from 0 to 10 wt %. The first row has SDS = 0 mM, the second 0.50 mM. In the third and fourth rows, the total surfactant concentration is 0.50 mM, but the composition of the surfactant mixtures is 75%:25% (anionic: nonionic). The last row has 0.5 mM C_12_E_6_. Photographs were taken approximately 2 days after preparation.

Mixing oppositely charged polymers and particles leads to complex phase separation, characterized by two discrete phases, one phase being opaque, and therefore very rich in large(r) structures, the second showing various degrees of opalescence due to the presence of dispersed nanoparticles. Figure 1a–d presents photographs of a large matrix of a series of samples in which the concentration of silica has been increased from 0 to 10 wt % in the presence of fixed amounts of polymer (typically 1000 ppm) at selected values of SDS concentration (0 ≤ [SDS] ≤ 1 mM). Our previous electrophoretic NMR characterization of the binary polymer/SDS systems indicates that the polymer/SDS solution phases separate (due to charge neutrality) around typically 4–8 mM for a polymer concentration C*_polymer_* = 1 wt %, suggesting that over the concentration window studied here (0–1 mM SDS), the polymer retains some cationic character, except perhaps at the higher SDS values studied [32]. Figure 2 presents a subset of these systems additionally in the presence of the nonionic surfactant, hexaethylene glycol monododecyl ether (C_12_E_6_). It should be noted that the uncharged polymer showed no measurable interaction with SDS. 

Traversing the row of samples from left to right, i.e., the only variable increasing being the silica concentration, the samples become increasingly opaque, with the volume of the opaque phase largely increasing. A similar pattern is observed in the uncharged and charged cases. Where SDS has been added, the volume of the opaque phase also depends on [SDS]. In some cases, the opaque phase is the supernatant, and in some cases the subnatant. As one moves vertically through the series, the same gross pattern is observed, but it is clear that the switch from super- to subnatant is displaced, and the phase volumes vary. 

Further interpretation of these visual phase diagrams is challenging, as the composition of each phase is unknown, and that of the opaque phase continues to change with time. Given the density of the silica, it is slightly surprising that the opaque phase creams at all. If one removes the dense phase, and allows it to stand, it will further phase separate, leading to additional volume of clear phase and more dense phases. Therefore, to accelerate this phase separation, the samples were gently centrifuged.

In order to understand the composition of the separated phases, the less dense (termed the equilibrium phase) has been characterized by both solvent relaxation and by dry weight analysis. Making the assumption that the bulk of the weight of the dried sample recorded in this manner arises from the particles, we therefore define this as an equilibrium particle concentration, and thus provides a convenient parameter (*x*-axis) on which to base solvent relaxation data, and thereby to enable in parallel the comparison with bare silica dispersions over the same initial concentration range. For the silica comparators, no differences between initial and equilibrium concentrations were observed in the dry weight analysis. The solvent relaxation in these no-polymer systems can therefore be compared with the with-polymer data.

**Figure 3:**
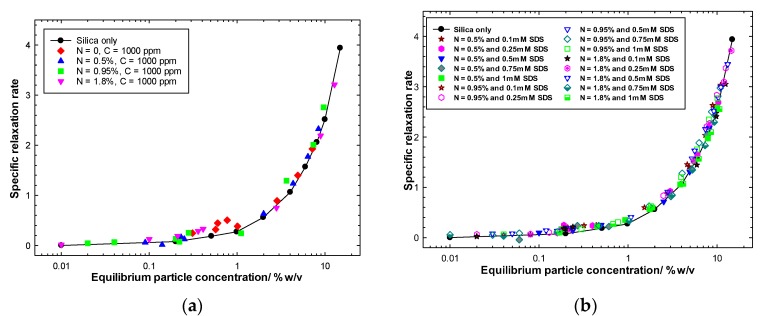
(**a**,**b**) Solvent-specific relaxation rate as a function of equilibrium particle concentration for all the cellulosic polymer/anionic surfactant/silica systems studied here; polymer concentration dependence when [SDS] = 0, (**a**); surfactant concentration dependence when [polymer] = 1000 ppm. The symbols refer to the series of experiments in Figure 1a–d and Figure 2, as per figure legend.

Solvent relaxation provides a convenient method to characterize any near-surface structures via the dynamics of the solvent molecules, in this case, water [33,34,35,36,37,38,39]. The relaxation rate is usually enhanced if a polymer layer is present, compared with the same surface area, due to the presence of a volume of trapped water. Figure 3 presents the solvent-specific relaxation rate, i.e., the rate normalized to pure water, plotted in terms of the equilibrium silica concentration. All samples studied here have been reproduced on this single figure. As may be seen, there is a universality to this curve that relates the measured specific solvent relaxation rate to the equilibrium silica concentration, this universality holding for particles that have been exposed to polymer, to surfactant and to mixtures of polymer and surfactant, as well as those that have not been exposed. 

Making the reasonable assumption that any added polymer or surfactant that adsorbs to the surface would perturb the solvent-specific relaxation rate [33,34,35,36,37,38,39], this universality strongly suggests that in all cases the particles detected in the solvent relaxation experiment are uncoated. In other words, the opaque phase consists of a concentration of polymer and particle with a composition to saturate that interaction, and any excess polymer or particle is displaced to the less-dense phase. When there is excess polymer present, all of the silica is removed, and one observes a solvent-specific relaxation rate that is characteristic of a simple polymer solution which is identical to the pure solvent, R_2sp_ tends to 0. When there is excess silica present, the polymer removes a fraction of the silica, and the solvent-specific relaxation rate is consistent with a bare silica dispersion commensurate with the remaining equilibrium concentration.

In those samples that also contain SDS, it is clear that the same universality between mass fraction and surface area exists. In this case, the anionic surfactant binds to the cationic polymer, thereby reducing the effective charge on the polymer, such that for a given polymer concentration, the polymer-surfactant complex removes less silica from the dispersion compared to the polymer alone. Nonetheless, all the detected silica surface is bare. In other words, in terms of the photographs in Figure 1 and Figure 2, addition of SDS moves the phase diagram to the left. Similarly, one can also estimate the concentration of SDS required to nullify the charge on the cationic polymer, and therefore “turning off” any adsorption to the anionic surface. 

Further confirmation of this simple binary phase separation may be sought by considering the zeta-potentials of the equilibrium (phase separated) silica phases (Table 1), these values being consistent with the original bare silica dispersion. 

**Figure 4:**
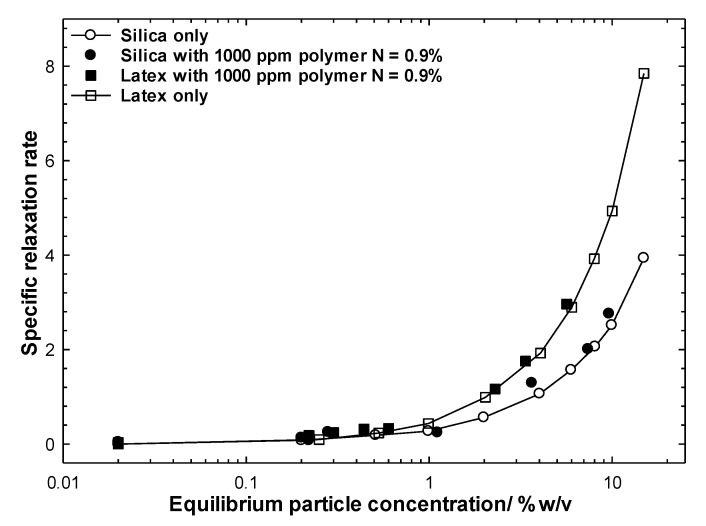
Solvent-specific relaxation rate as a function of equilibrium particle concentration for cationic polymer N = 0.95% at 1000 ppm for anionic silica (circles, open symbols no polymer, filled symbols with polymer) and anionic latex systems (squares, open symbols no polymer, filled symbols with polymer).

As an aside, a similar observation may be drawn from analogous studies using polystyrene-butadiene latex rather than silica (Figure 4). 

Consider the impact of addition of a nonionic surfactant to the polymer/particle/anionic system. The two surfactants would be expected to interact strongly and synergistically, and it is known that the presence of the nonionic often leads to an enhancement in the counterion dissociation of the anionic surfactant, effectively increasing the charge on the mixed surfactant complex. 

**Figure 5:**
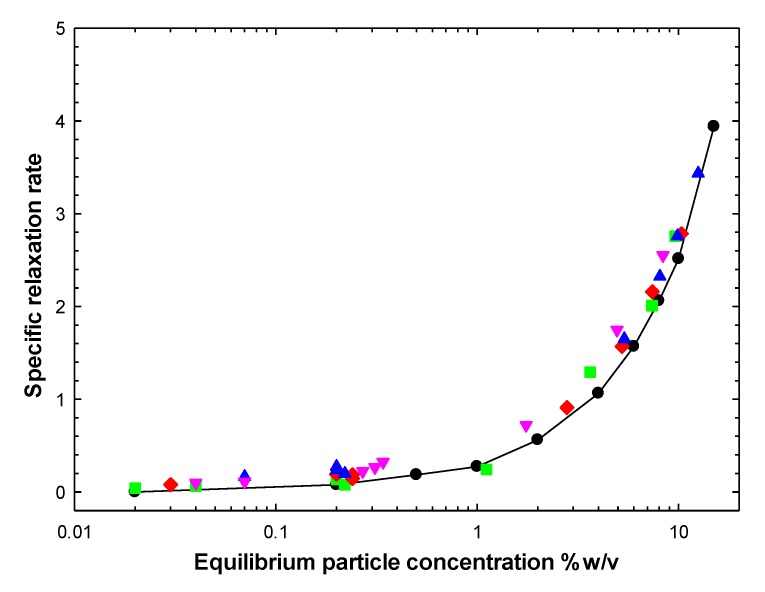
Solvent-specific relaxation rate as a function of equilibrium particle concentration for polymer N = 0.95% at 1000 ppm in the presence of anionic-nonionic surfactant blends; 0 (simple SDS) red diamonds, 0.75 blue triangles and 1.0 (simple nonionic) purple triangles. Additionally shown for comparison is the silica only dataset (black circles) and the SDS = 0 dataset. The total surfactant concentration is 0.5 mM.

Figure 5 presents the solvent-specific relaxation rate for a select set of experiments in which the cationic polymer has been first exposed to mixed anionic-nonionic (SDS-C_12_E_6_) surfactant blends, before being added to the silica dispersion. Phase separation still occurs, with the visual pattern not too dissimilar to that observed in the absence of the nonionic surfactant, and reminiscent of that observed at lower anionic surfactant concentrations. As may be seen, the same universality exists, between the dry weight analysis and the nature of the silica surface, implying the nonionic surfactant is merely a spectator in the polymer/SDS/particle interaction.

The uncharged (parent) polymer induces flocculation of the silica dispersion via a bridging mechanism, most likely through the hydroxyl groups. The grossly similar behavior of the three charged polymers indicates that the presence of the charge on the polymer leads to an increased capacity to remove silica, with the electrostatic interaction promoting the polymer bridging. Adding surfactant to the polymer solution induces complex formation through an associative interaction, which stoichiometrically reduces the prevailing charge on the polymer, and inter alia, its capacity to flocculate the silica. The uniformity of all these datasets, and the fact that all the surfaces detected in this manner are bare, indicates that the polymer-surfactant interaction must dominate the polymer-particle interaction, as found by Terada et al. [30], thus accounting for the lack of dependence of the phase diagram or solvent-specific relaxation rate on [SDS]. Two populations of particles exist in these slowly flocculating systems—bare (equilibrium phase) and fully/partially coated particles (dense phase), the balance of which is defined by the polyelectrolyte concentration, or more accurately, the concentration of oppositely charged polymer segments, the latter also defined by the presence of any added competing surfactant. Adding anionic surfactant that competes preferentially for the cationic charges on the polymer weakens the polymer-particle interaction. Accordingly, experimental protocols that focus on the particle behavior (e.g., electrophoretic mobility) might not adequately capture this bare/coated particle distribution.

## 4. Conclusions

Dispersions of anionic particles are often destabilized by the addition of cationic polymers. Here, it is shown that macroscopic phase separation may be induced through a bridging mechanism when cationic hydroxycellulose derivatives are added to silica (and latex) dispersions, with the high-molecular-weight polymer aggregating sufficient particles to neutralize its charge completely, leaving any excess particle surface uncoated, i.e., a coexisting dense phase of aggregated material and an equilibrium phase of bare particles. This charge-neutralization-driven adsorption leads to a simple universal relationship between the initial polymer concentration, the degree of cationic modification of the polymer and the mass (or more precisely, surface area) of silica removed from solution. The same universality exists when anionic surfactants are present, with the appropriate modulation of the phase behavior being understandable in terms of the stoichiometric binding of surfactant to polymer.

## Figures and Tables

**Table 1:** Zeta-potential analysis of the equilibrium phases obtained from the centrifugation of the two-phase silica/polymer/surfactant systems studied here. All measurements were conducted in triplicate.

Analysis of the Equilibrium Phase Extracted from the Following Samples	Zeta Potential/mV
Stock, as-supplied Ludox, 1-in-10 dilution	−40.4 (±1.8)
Dialysed Ludox	−35.5 (±1.5)
Dialysed Ludox plus polymer N = 0.5%	−30.9 (±1.3)
Dialysed Ludox plus polymer N = 0.95%	−30.8 (±1.7)
Dialysed Ludox plus polymer N = 1.8%	−31.4 (±2.4)
Dialysed Ludox plus polymer N = 0.5% and SDS	−31.4 (±1.6)
Dialysed Ludox plus polymer N = 0.95% and SDS	−29.9 (±2.2)
Dialysed Ludox plus polymer N = 1.8% and SDS	−32.6 (±2.8)
